# Transcriptome analysis of *Auricularia fibrillifera* fruit-body responses to drought stress and rehydration

**DOI:** 10.1186/s12864-021-08284-9

**Published:** 2022-01-15

**Authors:** Yiqin Wang, Zhifen Yang, Luxi Shi, Rui Yang, Hao Guo, Suqin Zhang, Guangdong Geng

**Affiliations:** College of Agriculture, Guizhou University, Guiyang, Guizhou China

**Keywords:** *Auricularia fibrillifera*, Transcriptome analysis, Drought stress, Rehydration, Melanin, Caffeine, Gibberellin, γ-aminobutyric acid

## Abstract

**Background:**

Drought stress severely restricts edible fungus production. The genus *Auricularia* has a rare drought tolerance, a rehydration capability, and is nutrient rich.

**Results:**

The key genes and metabolic pathways involved in drought-stress and rehydration were investigated using a transcriptome analysis to clarify the relevant molecular mechanisms. In total, 173.93 Mb clean reads, 26.09 Gb of data bulk, and 52,954 unigenes were obtained. Under drought-stress and rehydration conditions, 14,235 and 8539 differentially expressed genes, respectively, were detected. ‘Tyrosine metabolic’, ‘caffeine metabolism’, ‘ribosome’, ‘phagosome’, and ‘proline and arginine metabolism’, as well as ‘peroxisome’ and ‘mitogen-activated protein kinase signaling’ pathways, had major roles in *A. fibrillifera* responses to drought stress. ‘Tyrosine’ and ‘caffeine metabolism’ might reveal unknown mechanisms for the antioxidation of *A. fibrillifera* under drought-stress conditions. During the rehydration process, ‘diterpenoid biosynthesis’, ‘butanoate metabolism’, ‘C_5_-branched dibasic acid’, and ‘aflatoxin biosynthesis’ pathways were significantly enriched. Gibberellins and γ-aminobutyric acid were important in the recovery of *A. fibrillifera* growth after rehydration. Many genes related to antibiotics, vitamins, and other health-related ingredients were found in *A. fibrillifera*.

**Conclusion:**

These findings suggested that the candidate genes and metabolites involved in crucial biological pathways might regulate the drought tolerance or rehydration of *Auricularia*, shedding light on the corresponding mechanisms and providing new potential targets for the breeding and cultivation of drought-tolerant fungi.

**Supplementary Information:**

The online version contains supplementary material available at 10.1186/s12864-021-08284-9.

## Introduction

Drought is a severe environmental stress that negatively effects vegetative growth. The growth and yield of common organisms, including most edible fungi and terrestrial plants, suffer irrecoverable damage as a result of serious drought stress. Büntgen et al. (2015) found that the fruit body number of edible mushrooms, including *Boletus edulis* and *Lactarius* spp., decreased by three times due to the dry season period. In addition, the overall weight of fruit bodies has also declined since 1995, with a drop of nearly 10 tons [1].

Drought triggers organismal responses, including signal transduction, gene expression regulation, respiration enhancement, osmoprotectant and antioxidant enzyme accumulation, and growth inhibition [2]. Excessive reactive oxygen species (ROS) are produced under drought-stress conditions, thereby affecting redox homeostasis and producing oxidative stress, as evidenced by severe cellular damage as a result of peroxidation, reduced cell membrane stability, and increased protein denaturation [3–5]. The antioxidant systems that regulate ROS levels consist of both enzymatic, such as catalase (CAT), glutathione reductase, and peroxidase (POD), and non-enzymatic, such as ascorbate, glutathione, and ROS scavengers [6, 7]. Osmotic adjustment and cellular compatible solute accumulation are widely recognized to have roles in plant adaptation to dehydration, mainly through turgor maintenance and the protection of specific cellular functions by defined solutes [8]. A high proline content in pepper increases the leaf water content and reduces oxidative damage [9]. As the drought-stress level increases, the contents of proline and other osmolytes increase, which helps maintain the relative water content in corn plants [10].

Next-generation sequencing technologies provide high-throughput resources for gene expression profiling at the whole-genome level [11, 12]. Transcriptome analyses provide deep insights into global expression profiles under stress conditions, and they reveal valuable information about the responses of organisms to abiotic stresses [13–15]. At present, transcriptome technology has been used in the lichen-forming fungus *Endocarpon pusillum* [16], as well as melanized fungi [17], Arbuscular mycorrhizal fungi [18], *Piriformospora indica* [19], Arabidopsis [20], chickpea [21], cucumber [22], and rice [23, 24], to analyze the molecular responses to drought stress and elucidate drought-related adaptive mechanisms. The lichen-forming fungus isolated from the desert lichen *E. pusillum* is extremely drought resistant. Some pathways participate in the drought response in *E. pusillum*; the genes associated with calcium-mediated and ‘mitogen-activated protein kinase (MAPK) signaling’ pathways were differentially expressed, and are worthy of further investigation [16]. Arbuscular mycorrhization may possibly affect the adjustment capacity of common bean during water scarcity events. Chickpea produces 1624 differentially expressed genes (DEGs) under drought-stress conditions, such as putative mannitol dehydrogenase, serine hydroxymethyltransferase 4-like, heat shock protein-like, cytochrome P450 81E8-like, and galactinol-sucrose galactosyltransferase-like genes, which would be helpful for the investigation of candidate genes responsible for chickpea drought tolerance [25]. In drought-tolerant maize inbred seedlings, the DEGs related to amino acid biosynthesis, stress signal transduction, carbohydrate synthesis, and cell-wall remodeling, as well as the transcription factors play key roles in drought tolerance [26]. After treating *Amorpha fruticosa* seedlings with PEG-6000, most DEGs were up-regulated, and ‘cell wall’, ‘signal transduction’, and ‘hormonal regulation-related’ pathways were functionally enriched [27]. *Auricularia* is an extensively cultivated mushroom worldwide owing to its high nutritional, economic, and medicinal values [28]. It has anti-tumor, anti-inflammatory, hypoglycemic, hypolipidemic, cholesterol-lowering, and immunomodulatory properties [29–33]. The fruit bodies of *Auricularia* become dry and enter dormancy under drought-stress conditions. Once watered, dormancy is broken. The viability of *Auricularia* is rare in the eukaryotic community under arid conditions. Thus, the rare drought tolerance and rehydration capability of *Auricularia* make it an ideal species to explore adaptive mechanisms against drought stress [34].

In recent years, most studies on *Auricularia auricula-judae* have focused on the genetic diversity, taxonomy [35], laccase-related genes [28], medicinal properties [36–38], extraction techniques of polysaccharides [39, 40], and the mechanism of melanin accumulation under freezing conditions [41]. *Auricularia fibrillifera* has a rare drought tolerance and rehydration capability. At present, there are limited reports on transcriptome analyses of the responses to drought stress and rehydration in *A. fibrillifera*. Here, the key genes and metabolic pathways under drought-stress and rehydration conditions were investigated through a transcriptome analysis of *A. fibrillifera* fruiting bodies. The objectives were to understand the molecular response patterns under drought-stress and rehydration conditions, and to clarify the molecular mechanisms involved in drought tolerance and rehydration. The results provide a valuable reference for *Auricularia* breeding and its cultivation.

## Results

### Overview of the transcriptomic responses to drought stress and rehydration

A total of 12 cDNA libraries (two treatments and two controls with three replicates of each) were constructed using equal amounts of RNA extracted from the fruit bodies of *A. fibrillifera* under both drought-stress and rehydration conditions. We obtained 173.93 Mb clean reads and 26.09 Gb of data bulk, with an average GC content of 60.60% (Table 1). Then, 52,954 unigenes were generated from assembled high-quality reads, which encompassed 59,611,528 nucleotides. The unigene length ranged from 200 to 45,246 bp, with an average of 1125 bp and a scaffold N50 size of 1743 bp. Among these unigenes, 42.22, 15.31, and 5.79% were longer than 1000, 2000, and 3000 bp, respectively (Supplementary Fig. S1).

**Table 1:** Sequence statistics of the *A. fibrillifera* transcriptome

Total number of unigenes	52,954
Total clean reads (Mb)	173.93
Total clean bases (Gb)	26.09
Average GC content (%)	60.60
Total nucleotides of unigenes (bp)	59,611,528
Mean length of unigenes (bp)	1125
Largest unigenes (bp)	45,246
Smallest unigenes (bp)	200
Scaffold N50 size (bp)	1743

**Fig. 1:**
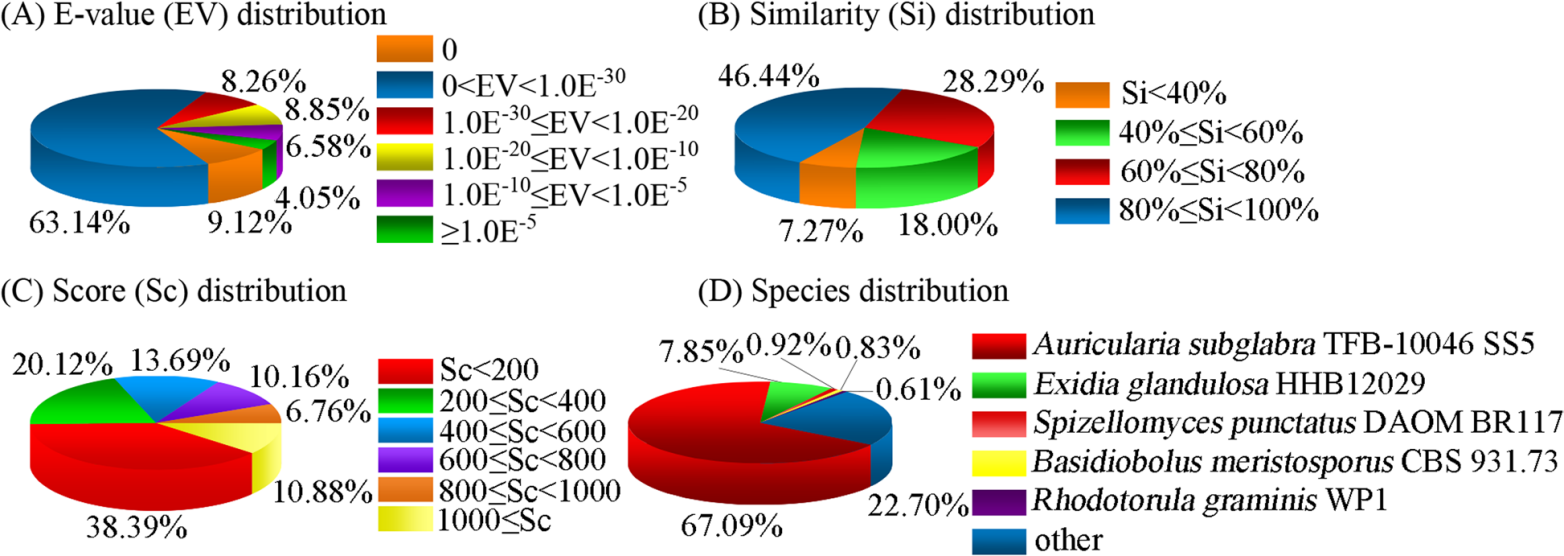
Characteristics of homology search among unigene sequences aligned by BlastX to the Non-redundant protein sequence database. **A** E-value distribution of BlastX hits for each unigene with an E-value threshold of 1.0E^− 5^. **B** Similarity distribution of the top BlastX hits for each unigene. **C** Score distribution of the top BlastX hits for each unigene. **D** Species distribution of the top BlastX hits for each unigene sequence with a cut-off E-value of 1.0E^− 5^

Using BlastX, the unigene sequences were annotated through searches against the public databases. A total of 52,954 unigenes had homologs in at least one of the public databases, including Non-redundant protein sequence database (NR), NCBI non-redundant nucleotide sequences (NT), Swiss-Prot protein sequence database (SwissProt), Eukaryotic Orthologous Groups (KOG), Kyoto Encyclopedia of Genes and Genomes (KEGG), Gene Ontology database (GO), and Pfam protein family database (Pfam) (Table 2). Among them, 42,149 (79.60%) and 31,877 (60.20%) unigenes have been annotated in the NR and Pfam databases, respectively. The unigenes annotated by the NR database were analyzed (Fig. 1). The E-value distribution revealed that 23.69% of the homologs varied from 1.0E^− 5^ to 1.0E^− 30^, while the E-value of most sequences (72.26%) was less than 1.0E^− 30^, which indicated strong homology (Fig. 1A). For the similarity distribution, 46.44% of the matches had 80 to 100% similarities as reported in the BlastX results, while 28.29% of the matches varied from 60 to 80% in similarity. Only 25.27% showed less than 60% similarities with the corresponding gene sequences (Fig. 1B). The score of 61.51% of the unigenes was ≥200, among which 10.88% presented values ≥1000 (Fig. 1C). *Auricularia subglabra* TFB-10046 SS5 exhibited the most BlastX matches (67.09%), followed by *Exidia glandulosa* HHB 12029 (7.85%) (Fig. 1D). In total, 37,530 coding sequences were detected using TransDcoder (Supplementary Table S1), and 4600 simple sequence repeat markers were distributed in 3627 unigenes.

**Table 2:** List of *A. fibrillifera* transcriptome annotations

Public database	No. of unigene hit	Percentage (%)
NR	42,149	79.60
NT	25,038	47.28
SwissProt	27,893	52.67
KOG	29,090	54.93
KEGG	30,506	57.61
GO	11,956	22.58
Pfam	31,877	60.20

*NR* Non-redundant protein sequence database, *NT* NCBI non-redundant nucleotide sequences, *SwissProt* Swiss-Prot protein sequence database, *KOG* Eukaryotic Orthologous Groups, *KEGG* Kyoto Encyclopedia of Genes and Genomes, *GO* Gene Ontology database, *Pfam* Pfam protein family database

### Identification of DEGs

**Fig. 2:**
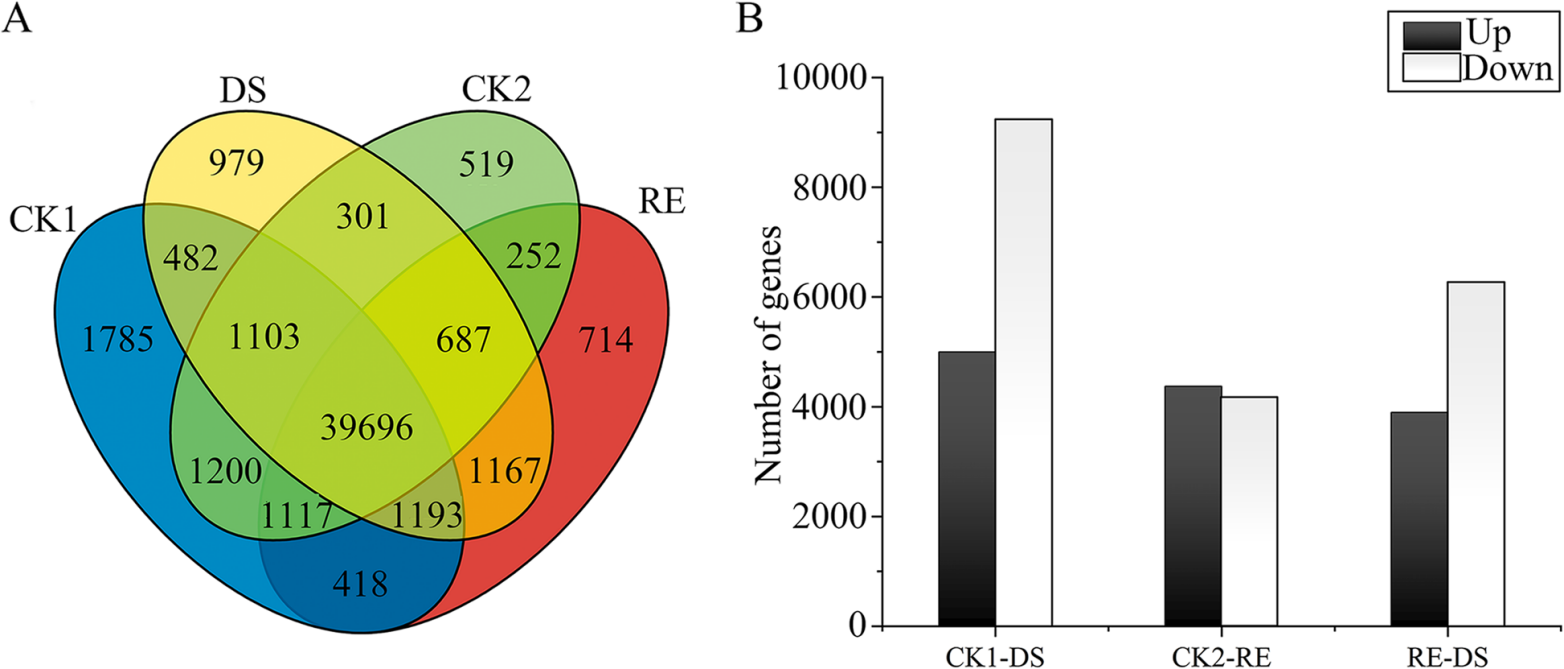
Number of DEGs in *A. fibrillifera* under drought-stress and rehydration conditions*.* **A** Venn diagrams of DEGs under drought-stress and rehydration conditions. **B** Number of up- and down-regulated DEGs under drought-stress and rehydration conditions. DS, drought stress; RE, rehydration; CK1 and CK2 were the controls of DS and RE, respectively. FDR ≤ 0.05 and |log_2_FC| ≥ 1 were used as the thresholds to judge the significance of gene expression differences

In total, 14,235 DEGs, 4996 up-regulated and 9239 down-regulated, were detected under drought-stress conditions (Fig. 2). During the rehydration process 8539 DEGs, 4373 up-regulated and 4166 down-regulated were found. There were 4703 common DEGs under drought-stress and rehydration conditions.

### DEG enrichment analyses of responses to drought stress and rehydration

A total of 1461/5 and 967/9 GO terms were enriched/significantly enriched under drought-stress and rehydration conditions, respectively. The structural categories of ‘ribosome’ and ‘ribosome and translation’ were significantly enriched under both drought-stress and rehydration conditions (Table 3). The up-regulated DEGs under drought stress were significantly enriched in ‘MAPK activity’ (0.26%), ‘mitochondrion’ (0.82%), ‘1-alkyl-2-acylglycerosphosphorine esterase activity’ (0.10%), ‘cytochrome-c oxidase activity’ (0.40%), ‘lipid binding’ (0.20%), and ‘fungal-type cell wall organization’ (0.18%) (Supplementary Table S2). The down-regulated DEGs were mainly enriched in ‘structural condition of ribosome’ (5.50%), ‘translation’ (5.09%), and ‘ribosome’ (4.07%). Ribosome function was inhibited, and the translation process in cells was reduced. During the rehydration process, DEGs were significantly up-regulated in ‘response to stress’ (0.59%), ‘MAPK activity’ (0.23%), and ‘transmembrane transporter activity’ (0.23%). The enriched terms for down-regulated DEGs were similar to those under drought-stress conditions. However, their percentages changed to 6.82, 6.34, and 5.23%, respectively.

**Table 3:** Significantly enriched GO terms under drought-stress and rehydration conditions

Stages	GO terms	Gene number	Q-value
Drought stress	Structural constituent of ribosome	726	6.88E^− 20^
	Translation	678	2.35E^− 16^
	Ribosome	533	3.78E^− 08^
	Cytosolic large ribosomal subunit	104	0.01
	Cytochrome-c oxidase activity	33	0.02
Rehydration	Translation	446	8.88E^−20^
	Structural constituent of ribosome	469	1.44E^−18^
	Ribosome	360	1.45E^−12^
	Translation elongation factor activity	55	0
	Transmembrane transporter activity	14	0
	Fungal-type cell wall	19	0
	Small ribosomal subunit	56	0.01
	Actin cytoskeleton	16	0.02
	Cell adhesion molecule binding	12	0.05

The significantly enriched KEGG pathways were determined to analyze the DEGs’ biological functions. There were 124 KEGG pathways involved in drought stress, among which ‘tyrosine metabolic’, ‘ribosome’, ‘phagosome’, and ‘aflatoxin biosynthesis’ pathways were significantly (Q-value ≤0.05) enriched (Table 4). Among these pathways, DEGs of ‘ribosome’, ‘amino sugar and nucleoside sugar metabolism’, and ‘MAPK signaling–yeast’ pathways accounted for 14.39, 10.45, and 10.11%, respectively. More than half of the DEGs in the ‘amino sugar and nucleoside sugar metabolic’ pathway were annotated as chitinase. In addition, many pathways related to sugar and amino acid synthesis were enriched, and 1449 DEGs were annotated to these pathways.

**Table 4:** Top 10 pathways enriched with differentially expressed genes under drought-stress and rehydration conditions

Stages	Pathways	Gene number	Q value
Drought stress	Ribosome	1295	2.13E^−53^
	Phagosome	167	2.24E^− 05^
	Aflatoxin biosynthesis	101	0.01
	Tyrosine metabolism	88	0.03
	Diterpenoid biosynthesis	32	0.08
	Caffeine metabolism	5	0.41
	Phenylalanine metabolism	58	0.41
	Phosphonate and phosphinate metabolism	8	0.41
	Butanoate metabolism	29	0.41
	Beta-alanine metabolism	68	0.44
Rehydration	Ribosome	770	2.30E^−27^
	Diterpenoid biosynthesis	27	0
	Aflatoxin biosynthesis	67	0
	Butanoate metabolism	25	0
	C_5_-Branched dibasic acid metabolism	6	0.01
	Cyanoamino acid metabolism	44	0.01
	Methane metabolism	56	0.04
	Fructose and mannose metabolism	34	0.12
	Tyrosine metabolism	53	0.12
	Thiamine metabolism	31	0.12

During the rehydration process, the DEGs were enriched in 122 KEGG pathways, with the main pathways being ‘diterpenoid biosynthesis’, ‘butanoate metabolism’, ‘C_5_-branched dibasic acid metabolism’, ‘ribosome’, ‘aflatoxin biosynthesis’, ‘cyanoamino acid metabolism’, and ‘methane metabolism’. Compared with drought stress, ‘diterpenoid biosynthesis’, ‘butanoate metabolism’, and ‘C_5_-branched dibasic acid metabolism’ pathways were specifically enriched. The number of up-regulated DEGs during the rehydration process was similar to that under drought-stress conditions. However, the numbers of down-regulated DEGs and DEGs per pathway during the rehydration process were approximately 50% the numbers found under drought-stress conditions. Many DEGs involved in the rehydration process were different from those activated under drought-stress conditions, indicating that the DEGs were pathway- and mechanism-specific.

Some enriched pathways, such as proline, were consistent with our physiological data, although they were not significantly enriched. Therefore, ‘tyrosine metabolism’, ‘caffeine metabolism’, ‘ribosome’, ‘phagosome’, ‘amino sugar and nucleoside sugar metabolism’, and ‘proline and arginine metabolism’, as well as ‘MAPK signaling pathway–yeast’ pathways might play significant roles in the drought tolerance of *A. fibrillifera*. ‘Dieterpenoid biosynthesis’, ‘butanoate metabolism’, and ‘C_5_-branched dibasic acid metabolism’ were significantly enriched during the rehydration process, which might be of paramount importance in the rehydration responses of *A. fibrillifera*.

### Expression levels of genes involved in responses to drought stress

#### Antioxidative substance-related genes

Melanin has antioxidant and free radical scavenging activities [42]. In this study, 88 DEGs were involved in the ‘tyrosine metabolic’ pathway. The greatest number of DEGs was annotated as tyrosinase (EC: 1.14.18.1) in this pathway, which plays a master role in catalyzing melanin synthesis. The level of tyrosinase expressed during the rehydration process was lower than under drought-stress conditions. The log_2_FC value of tyrosinase *CL504.Contig14_All* was the highest (4.71) under drought-stress conditions, but not after rehydration. *Unigene10928_All* (homogenesate1,2-dioxygenase (EC: 1.13.11.5) was highly down-regulated under drought-stress conditions, and this contributed to melanin synthesis. These genes may play vital roles in melanin accumulation, which alleviates the oxidative damage produced under drought stress conditions.

There were five DEGs in the ‘caffeine metabolic’ pathway that were annotated as members of the *CL2727* cluster. However, these five genes were all annotated to urate oxidase (EC: 1.7.3.3). The decrease in urate oxidase activity reduced the conversion of caffeine to 3,6,8-trimethyl-antoin, which improved caffeine accumulation under drought-stress conditions. On the contrary, most of these genes were up-regulated during the rehydration process.

The ‘peroxisome’ pathway is important in resisting oxidative stress, and superoxide dismutase (SOD) and catalase (CAT) are produced in this pathway. In total, 69 DEGs were annotated in the ‘peroxisome’ pathway under drought-stress conditions. *Unigene14272_All* (peroxin-13) was highly up-regulated under drought-stress conditions, but not after rehydration. Thus, antioxidants enhance the drought tolerance of *A. fibrillifera*.

#### Osmotic adjustment-related genes

Soluble sugars and amino acids have crucial roles in osmotic adjustment. A total of 1449 DEGs were annotated in pathways related to osmotic adjustment, such as ‘amino sugar and nucleotide sugar metabolism’, ‘fructose and mannose metabolism’, ‘mannose type O-glycan biosynthesis’, and ‘proline and arginine metabolism’ (Supplementary Table S3). There were 43 DEGs in the mannose pathway. Mannose performs an important role in osmotic adjustment [43]. ‘Mannose type O-glycan biosynthesis’ was significantly enriched, and the log_2_FC value of *CL8620.Contig2_All* reached 7.43 under drought-stress conditions. Additionally, four DEGs of the *CL2554* gene (dolichyl-phosphate-mannose-protein mannosyltransferase) cluster were annotated, and their log_2_FC values were > 5 under drought-stress conditions, but their expression was not observed after rehydration.

Proline plays a vital role in drought tolerance. In this study, 55 DEGs were annotated in the ‘proline and arginine metabolic’ pathway. *Unigene15963_All* in the ‘alanine, aspartate and glutamate metabolic’ pathway and *CL1716.Contig2_All* in the ‘arginine, proline and tryptophan metabolic’ pathway were highly expressed under drought-stress conditions, but not after rehydration. In particular, *CL1716.Contig2_All* played important roles in multiple amino acid and sugar metabolic pathways. On the contrary, *CL2495.Contig1_All* was down-regulated under drought-stress conditions, having a log_2_FC value of − 7.50. Thus, it should play negative roles in multiple amino acid metabolic pathways.

#### Genes in the ‘ribosome’ pathway

Ribosomes are responsible for translation. The GO and KEGG annotations determined that a large number of DEGs was annotated in the ‘ribosome’ pathway. Overall, 1295 DEGs were detected under drought stress conditions, among which 1046 presented |log_2_FC| values ≥2. Additionally, 770 DEGs during the rehydration process were enriched in the ‘ribosome’ pathway. Thus, more ‘ribosome’ pathway DEGs participated in the response to drought stress than to rehydration.

#### Genes in the ‘phagosome’ pathway

Foreign substances and damaged cells may be swallowed into phagosomes through actin endocytosis. Here, 23 actin beta/gamma 1 genes were involved in the ‘phagosome’ pathway, with the expression level of *CL118.Contig41_All* being the highest. In addition to regulating the intracellular ionic or pH balance, some ATPases drive numerous physiological or biological processes by ‘energy coupling’, including receptor-mediated endocytosis, intracellular membrane trafficking, protein degradation, and coupled transport [44]. Here, 12 vacuolar-type H^+^-ATPase (V-ATPase) genes, including *CL4156.Contig1_All*, *CL4156.Contig2_All* and *CL4156.Contig3_All*, were annotated in this pathway. These V-ATPases exist on phagocytic membranes and may help break down harmful substances.

#### Genes of the signaling pathways

The MAPK signal transmission network is important and has key roles in gene expression regulation and cytoplasmic functional activities. In total, 910 DEGs were annotated in the ‘MAPK signaling pathway-yeast’, and 282 were up-regulated under drought-stress conditions. There were 487 DEGs annotated as flocculation protein FLO11, 95 DEGs as mucin-19, and 408 DEGs as chitinase. Flocculation protein FLO11-related genes *Unigene104_All*, *CL6788.Contig3_All*, *CL3858.Contig3_All*, and *CL1874.Contig4_All* were highly expressed under drought-stress conditions, after which their levels decreased rapidly following rehydration. The differences in their log_2_FC values between drought-stress and rehydration conditions were > 9.

#### Chitinase-related genes

Hydrolases are involved in maintaining the plasticity of cell walls and may play roles in the branching and crosslinking of polymers [45]. A large number of the DEGs (907) were annotated as chitinases in the ‘amino sugar and nucleoside sugar metabolic’ pathway, with 306 up-regulated and 601 down-regulated DEGs being identified under drought-stress conditions. The log_2_FC values of 155 DEGs were > 2 under drought-stress conditions, and *Unigene104_All*, *Unigene5550_All*, *CL8307.Contig2_All* and *Unigene13034_All* were very sensitive to drought stress. They were abundantly expressed under drought-stress conditions, but not, or very lowly, expressed after rehydration*.*

### Expression of genes involved in responses to rehydration

#### Genes related to gibberellin (GA) synthesis

The ‘dieterpenoid biosynthesis’, ‘butanoate metabolism’, and ‘C_5_-branched dibasic acid metabolic’ pathways have important roles during the rehydration process. In total, 32 DEGs were annotated under the ‘diterpenoid biosynthetic’ pathway, and 27 were annotated during the rehydration process. Ent-kaurene oxidase catalyzes gibberellic aldehyde synthesis, which is a GA precursor. Ent-kaurene oxidase genes *CL264.Contig8_All* and *Unigene8480_All* were up-regulated during the rehydration process, indicating that GA plays major roles in breaking dormancy and improving growth after rehydration in *A. fibrillifera* (Supplementary Table S4).

#### The genes related to 4-aminobutyric acid (GABA) synthesis

GABA may act respond to osmotic pressure and alleviate water stress [46]. ‘Butanoate metabolism’ was significantly enriched during the rehydration process, and GABA was the main product of this pathway. There were 69 DEGs in the ‘butanoate metabolic’ pathway, among which 29 were annotated during the rehydration process. After rehydration, the log_2_FC value of *Unigene3125_All* (glutamate decarboxylase) reached 3.30, which was 4.74 fold greater than under drought-stress conditions. This gene plays an important role in the transformation of L-glutamate into GABA. It was up-regulated during the rehydration process, and this increased GABA synthesis.

#### The genes in ‘C_5_-branched dibasic acid metabolism’

Valine, leucine, and isoleucine are growth-promoting amino acids that act as carbon sources during starvation, and they can be integrated into the tricarboxylic acid (TCA) cycle to produce respiratory energy [47]. These amino acids are produced by ‘C_5_-branched dibasic acid metabolism’. In this study, six up-regulated DEGs were enriched in ‘C_5_-branched dibasic acid metabolism’ during the rehydration process. Among them, *CL4410.Contig1_All, CL4410.Contig2_All*, *CL6195.Contig1_All*, and *CL8753.Contig2_All* were annotated as acetolactate synthase I/II/III large subunit, and the others (*CL7598.Contig2_All* and *CL7598.Contig3_All*) as 3-isopropylmethylate dehydrogenase.

### Verification of DEGs by qPCR, protein expression levels, and physiological-biochemical data

In total, 11 DEGs closely related to drought stress were selected for the qPCR analysis. All the genes amplification levels, as assessed by qPCR, agreed well with the RNA-seq patterns, and the correlation between RNA-seq and qPCR was measured using scatter plot changes, which displayed a positive correlation coefficient (r = 0.9285, *p* = 1.8715E^− 6^; Table 5; Supplementary Fig. S2).

**Table 5:** Verification of RNA-seq results by qPCR

Gene ID	qPCR	RNA-seq
CL8627.Contig6_All	−0.14	−0.82
CL1983.Contig1_All	0.44	−0.05
CL456.Contig3_All	0.40	0.41
CL652.Contig1_All	0.65	0.49
CL6704.Contig1_All	1.78	0.91
CL4410.Contig1_All	1.37	0.97
CL2996.Contig9_All	1.09	1.29
CL118.Contig41_All	1.06	1.67
CL3812.Contig6_All	1.55	2.52
CL3209.Contig8_All	2.83	4.37
CL3274.Contig2_All	3.05	4.97
Unigene5564_All	5.30	1.68
R coefficient	0.9285	
*p*	1.8715E^− 6^	

*A. fibrillifera-*specific pathways, such as ‘tyrosine metabolism’, ‘caffeine metabolism’, ‘butanoate metabolism’, ‘biosynthesis of antibiotics’, and ‘phagosome’, were also enriched by the proteome analysis (Table 6). Many DEG-corresponding differentially expressed proteins (DEPs) were detected in these pathways, and the expression levels displayed by some DEGs were consistent with those of the DEPs. These data demonstrated that the transcriptomes accurately reflected the responses of *A. fibrillifera* to drought stress and rehydration.

**Table 6:** The expression levels of some specific DEGs/DEPs under drought-stress conditions in *A. fibrillifera*

Gene ID	Pathways	Function	log_2_FC	
			Gene	Protein
*Unigene219_All*	Tyrosine metabolism	Tyrosinase	3.41	2.36
*CL2727.Contig3_All*	Caffeine metabolism	Urate oxidase	−1.25	1.04
*Unigene10455_All*	Butanoate metabolism	Glutamate decarboxylase	−1.97	− 1.58
*CL4410.Contig1_All*		Acetolactate synthase activity	1.01	2.19
*CL1463.Contig1_All*	Biosynthesis of antibiotics	3-oxoacyl-[acyl-carrier protein] reductase	3.11	3.98
*Unigene5022_All*		Catalase	2.89	2.08
*Unigene431_All*		Sterol 22-desaturase	−1.53	−1.32
*CL5899.Contig1_All*	Phagosome	Ras-related protein Rab-5C	1.26	2.10

**Fig. 3:**
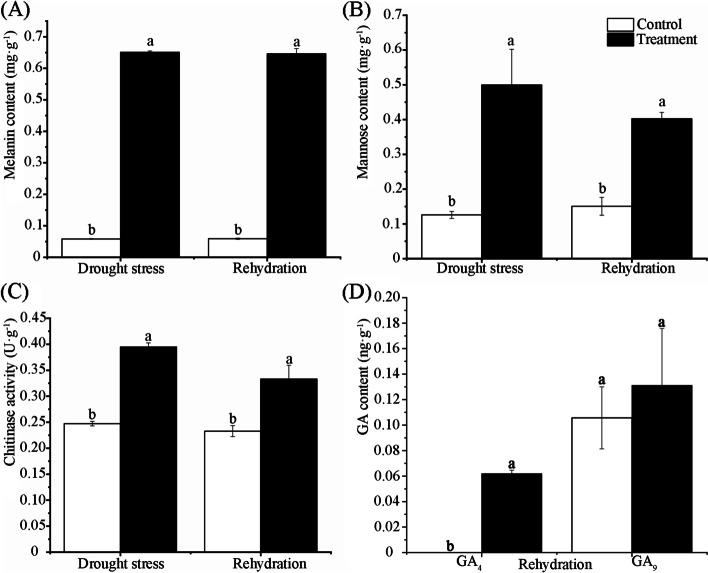
Effects of drought stress on the contents of melanin (**A**) and D-mannose (**B**), chitinase activity (**C**), and rehydration on the GA content (**D**) in *A. fibrillifera*. Bars show means ± SDs (*n* = 3). Values with different letters are significantly different at *p* < 0.05

The melanin content of fruiting bodies under drought stress conditions was 10.19-fold higher than that of the control (Fig. 3A). After rehydration, the melanin content decreased slightly. The difference in melanin content was not significant between drought stress and rehydration conditions, which may be attributed to the short rehydration time (only 1 h). D-mannose content and chitinase activities were significantly higher under drought stress than in the control. After rehydration, they started to decrease (Fig. 3B, C).

Nine kinds of GAs were detected during the rehydration process in *A. fibrillifera.* The contents of GA_4_ and GA_9_ were higher than those of the control, respectively (Fig. 3D). GA_4_ content showed significantly higher after rehydration than in the control. These physiological-biochemical results were consistent with the above transcriptome data.

## Discussion

### De novo sequencing and DEG analysis

Drought stress triggers a wide variety of fungal responses, ranging from molecular processes to physiological metabolism. In this study, 14,235 and 8539 DEGs were detected under drought-stress and rehydration conditions, respectively. In *Auricularia heimuer*, 7056 predicted proteins that accounted for 43.43% of the entire genome were mainly distributed in five functional entries, ‘binding’, ‘catalytic activity’, ‘cellular process’, ‘metabolic process’, and ‘single-organism process’ [48]. Similar results were obtained in this study. Most DEGs of *A. fibrillifera* were annotated as ‘binding’, ‘catalytic activity’, ‘cellular process’, ‘membrane part’, and ‘organelle part’.

### Transcriptome responses to drought stress

#### Antioxidative protection

The exposure of plants to certain environmental stresses quite often leads to ROS generation, which causes oxidative damage and impairs normal cellular functions [49]. The ROS increase at the early stage of drought stress induces the enzymatic and non-enzymatic scavenging systems to sequester ROS, so that the radical scavenging ability and strong drought tolerance was maintained in *Erythrodontium julaceum* mosses [50]. Melanins can protect fungi from abiotic stresses. They are endowed with a remarkable antioxidative activity and well-recognized photoprotective properties [51]. Melanin has antioxidant and free radical scavenging activities. The melanin produced by *Aureobasidium melanogenum* XJ5–1 obtained from the Taklimakan Desert can play an important role in the adaptation of yeast strains to drought stress treatments [52]. *A. pullulans* and *A. melanogenum* are black-yeast-like surface colonizers and are commonly encountered as contaminants in hospitals. These species are able to produce melanin, which plays a role in protection against environmental stress and irradiation [53]. Certain fungi can produce melanin via dihydroxyphenylalanine, which is used by tyrosinases or laccases to hydroxylate tyrosine to dopaquinone, which then auto-oxidizes and polymerizes, producing black polyphenolic heteropolymers [42]. Gray-green dihydroxynaphtalene-melanins establish the structural integrity of conidial cell walls and their adhesive properties in *Aspergillus fumigatus* [54]. Dopaquinone (ortho-quinone of 3,4-dihydroxyphenylalanine) is the precursor of melanin, and it is formed through the oxidation of L-tyrosine by the melanogenic tyrosinase [55]. In this work, most DEGs were involved in melanin synthesis in the ‘tyrosine’ pathway, and 24 DEGs were annotated as tyrosinase. These genes act as goalkeepers in every pathway of melanin synthesis. The expression levels of these DEGs under drought-stress conditions were significantly greater than those after rehydration. The highest log_2_FC value of these DEGs reached 4.71 under drought-stress conditions. Homogentic acid 1,2-dioxygenase negatively regulates the synthesis of melanin [56]. Here, a highly down-regulated homogentic acid 1,2-dioxygenase gene (*Unigene10928_All*) was also found. The melanin content of fruiting bodies under drought stress conditions was 10.19-fold higher than that of the control (Fig. 3A). These various DEGs work together to promote melanin accumulation.

Caffeine, a xanthine alkaloid, is an active ingredient in coffee and present in many foods and beverages. Caffeine is an effective antioxidant that can eliminate ·OH, and its antioxidant ability is similar to that of glutathione, and significantly higher than that of ascorbic acid [57, 58]. In this experiment, five DEGs (enrichment ratio = 0.71) were expressed in the ‘caffeine metabolic’ pathway under drought-stress conditions. They were all annotated as urate oxidase. However, they were not detected after rehydration. Thus, caffeine might perform crucial roles in *A. fibrillifera*’s adaptation to drought stress.

The ‘peroxisome’, ‘ascorbate’, and ‘aldarate metabolic’ pathways were identified in this work. A total of 79 DEGs were annotated in the ‘peroxisome’ pathway, of which 69 were annotated under drought-stress conditions. CAT and SOD participate in hydrogen peroxide metabolism in the peroxisomal targeting sequence 1 system. *Unigene14272_All* (peroxin-13) was up-regulated under drought-stress conditions, but not after rehydration. The levels of glutathione reductase, glutathione peroxidase, and glutathione S-transferase increase during slow drying or during rehydration following rapid drying in the drought-tolerant moss *Tortula ruralis* [59]. NADPH plays important roles in maintaining the reduced glutathione content in cells [60]. In total, 39 DEGs in the ‘glutathione metabolic’ pathway were annotated under drought-stress conditions. *CL8514.Contig2_All* (glutathione peroxidase) was up-regulated under drought-stress conditions, but not after rehydration. Thus, these antioxidant enzymes may scavenge harmful ROS and free radicals to protect cell membranes and proteins under drought stress conditions in *A. fibrillifera*.

Sixty-three up-regulated DEGs were annotated in the ‘aflatoxin biosynthesis’ pathway under drought conditions. However, the expression levels of most DEGs (74.60%) decreased rapidly at 1 h after rehydration. Aflatoxin may participate in *A. fibrillifera*’s responses to drought stress by reducing oxidative damage [61].

#### Osmotic adjustment

In response to abiotic stresses, amino acid metabolism plays an important regulatory role because amino acids act as potential regulatory and signaling molecules, as well as precursors for energy-associated metabolites and numerous secondary metabolites [49]. In this study, *CL1716.Contig2_All* (amidase) enhanced GABA synthesis, and it was highly expressed (log_2_FC = 7.72) under drought-stress conditions, but not after rehydration. It also participated in other amino acid metabolic pathways, such as ‘tryptophan and phenylalanine metabolisms’. *CL2495.Contig1_All*, an aldehyde dehydrogenase (NAD^+^) gene, was annotated in multiple amino acid metabolic pathways, including ‘alanine, aspartate and glutamate metabolism’, as well as in ‘butanoate metabolism’. Proline is the amino acid most associated with stress-related responses in plants. Its accumulation varies among species, but it can be 100-times greater under stress conditions compared with control conditions [62]. Proline synthesis appears to be the preferred method for withstanding prolonged osmotic stress in *Jeotgalibacillus malaysiensis* [63]. In this study, the ‘proline and arginine metabolic’ pathway was enriched under drought-stress conditions. There were 16 up- and 38 down-regulated DEGs under drought-stress conditions. Proline iminopeptidase is a constitutive enzyme that digests a large number of n-proline copolymers to form proline [64]. In total, seven DEGs were annotated as proline iminopeptidase, and their expression levels were significantly greater under drought-stress conditions than after rehydration. These DEGs promoted proline accumulation under drought-stress conditions. This was consistent with the increased proline content under drought-stress conditions in the physiological experiment (unpublished data).

A water deficit during drought stress inevitably leads to osmotic stress. Under osmotic-stress conditions, the accumulations of sugars and amino acids, as well as K^+^ and glycine betaine, help organisms to resist osmotic stress [65]. *Staphylococcus xylosus* responds to salt stress by overexpressing genes related to the synthesis of osmotic protectants [66]. Here, 10 pathways related to osmotic adjustment, such as ‘fructose and mannose metabolism’, and ‘amino acid metabolic pathways’, were identified. *CL8620.Contig2_All* in ‘mannose type O-glycan biosynthesis’ was highly expressed under drought-stress conditions. D-mannose content was significantly higher under drought stress than in the control. After rehydration, it started to decrease (Fig. 3B).

#### Ribosome

Ribosomes are the centers of translation, which is the first step in the synthesis of many regulatory substances. Importantly, the regulation of the elongation phase of translation is broadly utilized in response to cellular stress, and is impacted by various mechanisms, including post-translational modifications of elongation factors [67] and ribosomes [68–70]. Most of the DEGs were enriched in the ‘ribosome’ pathway. In total, 1295 DEGs were detected under drought stress conditions, among which 1046 presented |log_2_FC| values ≥2. Additionally, 770 DEGs during the rehydration process were enriched in the ‘ribosome’ pathway. Thus, more ‘ribosome’ pathway DEGs participated in the response to drought stress than to rehydration.

#### Phagosome

Phagosomes are key organelles responsible for the innate ability of macrophages to participate in clearing apoptotic cells and maintain overall homeostasis [71]. ‘Phagosome’ pathway is significantly affected by drought in wheat [72]. This pathway requires the activity of a V-ATPase, and is induced by osmotic imbalances within endolysosomal compartments [73]. In this work, the ‘phagosome’ pathway was significantly enriched under drought-stress conditions. There were 23 DEGs in this pathway, among which *CL118.Contig41_All* had the highest expression level. The *CL118* gene cluster was annotated as actin beta/gamma 1, which is involved in F-actin-controlled phagocytosis.

#### Signal transduction

In all the fungal species, the ‘MAPK’ pathway plays important role in their physiology and development, including responses to different stresses, resistance to temperature changes, cell wall assembly and integrity, cell-cell signaling, and responses to damage-associated molecular patterns [74]. The central signal transduction pathway of *A. fumigatus* during hyperosmotic stress is the high osmolarity glycerol MAPK cascade [75]. Some MAPK cascade genes are differentially expressed in cotton under abiotic stress conditions, after plant hormone treatments and especially under drought-stress conditions [39]. Drought stress induces MAPK cascade responses in cotton, and when the cotton MAPK kinase gene *ghmkk3* is overexpressed in tobacco, drought tolerance is enhanced [76]. In this experiment, 282 up-regulated and 627 down-regulated DEGs in ‘MAPK signaling pathway-yeast’ were identified under drought-stress conditions. *Unigene104_All* (chitinase), *CL1874.Contig4_All* (signaling mucin HKR1), *Unigene13034_All* (chitinase), and *Unigene14521_All* (flocculation protein FLO11) were highly expressed under drought-stress conditions, but not, or lowly, expressed after rehydration.

Many stresses trigger transient increases in minor phospholipids, such as phosphoinositides and phosphatidic acid [77]. In this study, 37 DEGs were annotated in the ‘phosphatidylinositol signaling’ pathway. Among them, the three calmodulin genes, *Unigene2205_All, Unigene7676_All* and *Unigene5387_All*, had very obvious responses to drought stress and rewatering. They were significantly up-regulated under drought-stress conditions, but significantly down-regulated after rehydration. Their largest log_2_FC difference reached 12.89. In addition, the *Unigene9338_All* (1-phosphatidylinositol-4-phosphate 5-kinase) showed the highest expression in this pathway, which is involved in the synthesis of phosphatidylinositol 4-phosphate to phosphatidylinositol 4,5-bisphosphate. As a result, MAPK and phosphatidylinositol signaling system pathways may participate in *A. fibrillifera*’s responses to drought stress and rewatering, and then lead to drought-tolerant effects on physiology and growth.

#### Chitinase

Fungal chitinases play an important role not only in degradation, but also in morphogenesis, cell division, autolysis, and acquisition of chitin [45]. *PcchiB1* in *Penicillium chrysogenum*, which potentially encodes a class V chitinase, is responsible for cell wall integrity. The knockout strains showed distinct mycelial growth, but control strains presented highly fragmented and dispersed hyphae with the formation of only a few small-fringed pellets. In addition, the increase of PcchiB1 transcript levels led to the degradation of chitin in the fungal cell wall, which provided new nutritional resources for fungi [78]. The extending native stipe walls of *Coprinopsis cinerea* were associated with the release of N-acetylglucosamine and chitinases, especially ChiE1 and CHIII. These two chitinases reconstituted the wall extension of the heat-inactivated stipe, and their double knockdown reduced stipe elongation, mycelium growth, and wall extension [79]. The overexpression of chitinases in transgenic plants provides resistance to abiotic and biotic stresses [80]. In this work, it was shown that the fruiting body shrank and became hard under drought stress conditions (Supplementary Fig. S3); once watered, it would absorb water quickly and become soft and stretched. All chitinase genes were annotated as the chitinases [EC:3.2.1.14] by KEGG analysis. 306 up-regulated and 601 down-regulated DEGs were identified under drought-stress conditions, and the log_2_FC values of many chitinase related DEGs were very high, with the value of *Unigene104_All* being the greatest (10.15). Chitinase activities were significantly higher under drought stress than in the control (Fig. 3C). A total of 73 DEGs were grouped into seven GO terms related to cell wall, among which 19 were annotated as belonging to the chitinase [EC: 3.2.1.14] type and 50 as various proteins. Therefore, many DEGs related to chitinase may activate cell wall metabolic pathways and remodeling in order to adapt to drought stress in *A. fibrillifera*. We will focus on their classification, functions, and mechanisms in future research.

The main function of abscisic acid (ABA) is to regulate the plant’s water balance and osmotic stress tolerance [81]. However, in this research, the pathways and genes associated with ABA synthesis or metabolism were not found. ABA occurs mainly in vascular plants, such as angiosperms, gymnosperms, and ferns. Therefore, unlike in plants, ABA did not play key roles in *A. fibrillifera*’s responses to drought stress*.*

### Transcriptome responses to rehydration

#### GA synthesis

GA is a diterpenoid and an important phytohormone that promotes organ expansion and developmental changes. To date, the GA synthetic pathway in fungi is a mevalonate pathway. In this study, ‘dieterpenoid biosynthesis’ pathway was significantly enriched during the rehydration process, in which all the DEGs were annotated to GA synthesis. *CL264.Contig8_All* is an ent-kaurene oxidase that was up-regulated after rehydration, but down-regulated under drought-stress conditions. In contrast, *CL5200.Contig3_All* is a GA 2 beta dioxygenase that was down-regulated after rehydration, but up-regulated under drought-stress conditions. The two genes produce GA during the rehydration process, but degrade GA under drought-stress conditions. GA_4_ content showed significantly higher after rehydration than in the control. Thus, GA_4_ may break dormancy and promote cell division and growth during the rehydration process in *A. fibrillifera*.

#### GABA synthesis

GABA is a non-protein amino acid that is widely distributed throughout the biological world and is a suitable nitrogen source for many fungi [82]. In plants, GABA has multiple functions under non-stressed and stressed conditions. Exogenous applications of GABA confer greater stress tolerance by modulating the expression of genes involved in plant signaling, transcriptional regulation, hormone biosynthesis, ROS production, and polyamine metabolism [83]. Exogenous GABA effectively alleviates growth inhibition caused by adverse environmental conditions (e.g., drought, salt, extreme temperature, water, light, or oxygen stress) [84, 85]. Exogenous GABA improves sporulation in *Stagonospora nodorum* and *Botryosphaeria* sp. (class Dothideomycetes) [86]. Here, ‘butanoate metabolism’ was significantly enriched during the rehydration process, and its main product is GABA. *Unigene16234_All* and *Unigene3125_All* were annotated as glutamate decarboxylase (EC: 4.1.1.15) that convert L-glutamate into GABA, which had a very high log_2_FC value. Therefore, GABA may contribute to the sporulation and rapid growth recovery of *A. fibrillifera* after rehydration.

#### C_5_-dicarboxylic acid metabolism

In the ‘C_5_-dicarboxylic acid metabolism’ pathway, the synthesis of valine, leucine, and isoleucine is activated. Valine, leucine, and isoleucine are branched chain amino acids that are used as carbon sources to promote growth. The carbon skeletons of branched chain amino acids are generally converted to precursors or intermediates of the TCA cycle. The oxidation of leucine, isoleucine, valine, and lysine directly feeds electrons into the mitochondrial electron transport chain [47, 87, 88]. Because the ATP production of leucine, isoleucine, and valine is particularly high, the amounts of ATP produced by phosphorylation at the substrate level in the TCA cycle are increased [89]. In this study, six up-regulated DEGs were annotated in the ‘C_5_-dicarboxylic acid metabolism’ pathway during the rehydration process. They might provide energy and carbon skeletons for growth recovery after rehydration.

### Health care components in *A. fibrillifera* fruit bodies

In this study, we found many genes related to antibiotics, vitamins, and other health-related ingredients in *A. fibrillifera*. Diterpenoids are found mainly in fungi and plants, and they possess a wide spectrum of important biological activities, such as anti-inflammatory, anti-cancer, and anti-parasitic [90]. In total, 105 DEGs related to diterpenoids were identified in the transcriptome analysis. There were 3734 DEGs involved in the biosynthesis of antibiotics, such as ‘penicillin and cephalosporin biosynthesis’ (63 DEGs) and ‘carbapenem biosynthesis’ (16 DEGs). In total, 280 DEGs were annotated to ‘thiamine metabolism’, mainly in ‘vitamin B_6_ metabolism’ (68 DEGs). Thus, *A. fibrillifera* is rich in human health care-related substances, which are worthy of further exploration.

## Conclusion

In total, 14,235 and 8539 DEGs were detected under drought-stress conditions and during the rehydration process, respectively. ‘Tyrosine metabolism’, **‘**caffeine metabolism’, **‘**ribosome’, ‘phagosome’, and ‘proline and arginine metabolism’, as well as **‘**peroxisome’ and ‘MAPK signaling pathway-yeast’ pathways, were enriched under drought stress conditions. The strong drought tolerance of *A. fibrillifera* may be mainly attributed to ROS scavenging, osmoregulation, signal transduction, cell wall remodeling, and phagocytosis. GA_4_ and GABA might promote the rapid recovery of growth after rehydration. Some important candidate genes (such as *Unigene219_All*, *CL2727.Contig3_All*, *Unigene10455_All*, *CL4410.Contig1_All*, and *CL1463.Contig1_All*) related to drought stress and rehydration were also found. Elucidating the function of these drought/rehydration-related genes will be valuable for genetic modification in *Auricularia*. Finally, many genes related to antibiotics, vitamins, and other health-related ingredients were found in *A. fibrillifera*, which is a nutritious food for humans. The outcomes of proteomic and physiological-biochemical analyses were consistent with the transcriptome data. These results shed light on the mechanisms of drought tolerance and rehydration, and they provide a valuable reference for *Auricularia* breeding and its cultivation. Further, the RNA-Seq data in the public repository have a wide variety of applications and accelerate the exploration in *Auricularia* researches.

## Materials and methods

### Material, growth conditions, and sampling

The fruiting bodies of drought-tolerant *Auricularia fibrillifera* cultivar ‘CSLZ’ was used for this experiment. This strain was obtained from tissue isolation, it was maintained in a potato-dextrose agar (PDA) medium at 4 °C, and was inoculated into the culture medium to produce the fruiting bodies. Its culture medium consisted of 83% sawdust, 14% wheat bran, 1% bean bran, 1% gypsum, and 1% lime. In total, 1 kg of mixed substrate was placed in heat-resistant polyethylene bags and then autoclaved for 1.5 h at 121 °C. The sterilized medium was inoculated with 0.5% grain spawn and maintained at 25 °C in the dark. When the culture medium was fully colonized by mycelia, the polyethylene bags were removed, and the substrate was placed in a culture room (25 ± 1 °C, 15-h naturally scattered light/9-h dark). The bags were sprayed regularly with water, approximately eight times per day, with 15 mL of water per bag of substrate. When the diameters of the fruit bodies reached 2 to 3 cm, drought-stress treatments were started, and the fruit bodies on the substrate dehydrated naturally. Regularly watered fruit bodies on the substrate were used as controls. When the water loss rate of fruit bodies reached 60% (DS) compared with its respective control (CK1), uniformly sized fruit bodies were collected as the first samples. After, the fruit bodies on the substrate were rewatered regularly. The second sampling was performed when the water loss rate of fruit bodies was 50% (RE, 1 h after rehydration) compared with its respective control (CK2). These sampling stages were chosen on the basis of phenotypic and physiological data. Each sample pool included 15 individual fruit bodies, and three biological replications were employed in this experiment. All the samples were immediately frozen in liquid nitrogen after sampling and stored at − 80 °C for further use.

### Total RNA extraction, cDNA library construction, and sequencing

Total RNA was extracted using a TRIzol® reagent kit (Tiangen Biotech, Beijing, China) in accordance with the manufacturer’s instructions, and then treated with TaKaRa RNase-free DNase I for 30 min. The quality and quantity of total RNA were estimated using a NanoDrop TM (Thermo Fisher Scientific, Waltham, MA, USA). cDNA library construction was accomplished using cDNA library construction Kit (Beijing Genomics Institute, Shenzhen, China), and then, the libraries were subjected to paired-end read sequencing on the BGISEQ-500 platform at the Beijing Genomics Institute. Briefly, 20 μg of total RNA per sample was mRNA enriched [enrichment of mRNA containing Poly(A) tails by magnetic beads with oligo dT] or underwent rRNA removal (hybridization of rRNA with a DNA probe). After that, the mRNA was randomly fragmented in fragmentation buffer, transcribed with random N6 primers, and double-stranded cDNA was then synthesized. The end of the synthesized double-stranded DNA was flattened and phosphorylated at the 5′ end. The 3′ end was sticky and protruded from an “A”, which was connected with a joint protruding from a “T”. The linkage products were amplified by PCR using specific primers. The PCR product was heat denatured into a single strand, and then, the single-strand DNA library was obtained by circularizing the single-strand DNA with a bridge primer. Then, high-throughput sequencing was performed on the BGISEQ-500 platform at the Beijing Genomics Institute.

### RNA-sequencing (RNA-seq) data processing and de novo assembly

The raw paired-end reads were cleaned by removing adapter sequences, empty reads, and low-quality reads (reads with > 5% unknown base pairs ‘N’) using SOAPnuke (Beijing Genomics Institute, version: 1.4.0, parameter: -l5, −q 0.5, −n 0.1) and trimmomatic software (version: 0.36, parameter: Illuminaclip:2:30:10, Leading:3, Trailing:3, Slidingwindow:4:15, Minlen:50) [91, 92]. Because there was no reference genome, Trinity de novo transcriptome assembly software was used for the transcriptome assembly of RNA-seq data from *A. fibrillifera* [93].

### Sequence annotation and classification

Using BlastX, the sequences were searched against the NCBI non-redundant (NR) protein database with a cut-off E-value of 10^− 5^ for annotation [94]. Gene Ontology (GO) terms were extracted from the annotation of high scoring BLAST matches in the NCBI NR protein database (E-value ≤1.0 × 10^− 5^, http://www.ncbi.nlm.nih.gov) using Blast2go (version: 2.5.0, parameter: default) [95], after which the GO categories were sorted using in-house perl scripts [96]. The annotations of Kyoto Encyclopedia of Genes and Genomes (KEGG, http://www.genome.jp/kegg) pathways were carried out using Blastall software (version: 2.2.23, parameter: default) against the KEGG database [97].

### Gene expression analysis

Using Bowtie, each RNA-seq library was individually aligned to the produced transcriptome assembly [98]. The RSEM package was used for the counting of alignments [99, 100]. The R Bioconductor package (EdgeR) was adopted to analyze DEGs [101]. The *P*-value established the threshold to test the differential gene expression. The P-value threshold for multiple tests was decided through the false discovery rate (FDR) [102]. The DEGs were selected on the basis of an FDR ≤ 0.05 and absolute log_2_fold change value (|log_2_FC|) ≥ 1 [103]. Using the KEGG Orthology Based Annotation System, the DEGs were mapped to the terms in the KEGG database for the pathway enrichment analysis [104]. Significantly enriched pathways related to DEGs were determined using the standard of corrected P-value (Q-value) ≤ 0.05.

### Quantitative RT-PCR (qPCR) analysis

For verification by qPCR, 11 key candidate genes were chosen based on their important functions associated with drought tolerance and high |log_2_FC| values (Supplementary Fig. S2). The same RNA samples used for the transcriptome analysis were reverse-transcribed using Power SYBR® Green PCR Master Mix (Thermo Fisher Scientific) with polyT primers. Primer 5 was used to design quantitative primers, which are listed in Supplementary Table S5. *GAPDH* was used as the internal control [105]. The 20 μL reaction mixture contained 10 μL of 2× Talent qPCR PreMix (Tiangen Biotech), 0.3 μM of each primer, 50 ng of cDNA as template, and RNase-free ddH_2_O, which was added to reach the final volume. qPCR amplification was performed on an ABI stepone Real-Time PCR System (Applied Biosystems, Fostery, CA, USA) using primers listed in Supplementary Table S5. The following PCR conditions were set: 3 min of preincubation at 95 °C, followed by 40 cycles for 10 s at 95 °C, 20 s at 59 °C, 20 s at 72 °C, and 5 s at 75 °C. Then, a DNA melting curve was produced by increasing the heat from 65 to 95 °C (with a rate of 0.5 °C and continuous fluorescence measurement) to confirm the specificity of the PCR products. The relative expression level was calculated using the 2^−ΔΔCt^ method with three biological replications and three technical replications [106].

### Protein expression level analysis

Important genes were indicated by the relative expression levels of the DEPs. Total proteins were extracted from the fruit bodies using the phenol method [107]. Trypsin [protein:trypsin = 40:1 (w/w)] was added for enzymolysis, followed by desalination and vacuum drying. The peptide samples were separated using an UltiMate 3000 UHPLC (Thermo Fisher Scientific), ionized by a nanoESI source, and then placed into a Q-Ex Active HF tandem mass spectrometer (MS, Thermo Fisher Scientific) for data-independent acquisition mode detection. The spectral library was constructed using the data-independent acquisition of targeted samples. Spectronaut was used to effectively de-convolute, accurately identify, and quantitatively analyze the data [108].

### Physiological-biochemical assay

Melanin was extracted based on a slightly modified version of Liu’ s method [109]. In short, 1.00 g of fruit body was fully ground in 50 mL of 1 M NaOH. The samples were treated in an ultrasonic cleaner (300 W) for 2 h at 60 °C. The supernatant pH was adjusted to 1.5 with 3 M HCL, and the supernatant was immersed in a boiling water bath for 10 h, and then centrifuged at 9156 *g* for 15 min. After the precipitation was cooled and dried, the melanin content was calculated.

The assay kits for the measurement of D-mannose (Cas No.: G0583W, Grace, Suzhou, China) content and chitinase (Cas No.: BC0825, Solarbio, Beijing, China) activity were applied in accordance with the manufacturer’s instructions.

GA content was detected using a slightly modified version of Deng’ s method [110]. In brief, 50.0 mg of fruit body was ground into powder, and dissolved in 500 μL acetonitrile/H_2_O (90:10, V/V). 10 μL of internal standard mixed solution (100 ng/mL) was added to the extract as internal standard for quantification. The mixture was rotated for 15 min, and then centrifuged at 12,000 *g* for 10 min at 4 °C. 10 μL of triethylamine and 10 μL of 3-bromopropyltrimethylammonium bromide were added to the resulting solution. The reaction solution was rotated, incubated for 1 h at 90 °C, evaporated to dryness under nitrogen flow, then redissolved in 100 μL acetonitrile /H_2_O (90:10, V/V) and passed through a 0.22 μM membrane filter for further LC (liquid chromatography)-MS/MS analysis. The sample extracts were analyzed using an UPLC (Ultra performance liquid chromatography)-MS/MS system (UPLC, ExionLC™ AD (AB SCIEX, Dublin, CA, USA); MS, Applied Biosystems 6500 Triple Quadrupole (Applied Biosystems). The analytical conditions were as follows: LC: column, Waters ACQUITY UPLC HSS T3 (100 mm × 2.1 mm i.d., 1.8 μm); solvent system, water with 0.04% acetic acid (A), acetonitrile with 0.04% acetic acid (B); gradient program, started at 5% B (0 min), increased to 95% B (10.0 min), 95% B (10.1–11.0 min), finally ramped back to 5% B (11.1 min), 5% B (11.1–14.0 min); flow rate, 0.35 mL/min; temperature, 40 °C; and injection volume, 10 μL.

### Data analysis

The data were analyzed in SPSS 21.0 (IBM Corp., Armonk, NY, USA). Duncan’s multiple range test was performed to determine differences between means at a significance level of *p* < 0.05, after significant effects were observed during an ANOVA. Pearson’s correlation analysis of binary variables was performed, and two variables were considered significantly correlated at *p* < 0.05.

## Supplementary Information

**Additional file 1.**

**Additional file 2.**

## Data Availability

All data generated or analyzed for this study are included in this article and its supplementary files. The raw sequence reads were deposited into NCBI SRA database under accession no. PRJNA695780 (https://www.ncbi.nlm.nih.gov/sra/?term=PRJNA695780).
