# Supplementary material for: Transcriptome analysis of Auricularia fibrillifera fruit-body responses to drought stress and rehydration
Source: BMC Genomics. 2022 Jan 15;23:58. doi: 10.1186/s12864-021-08284-9 (PMC8760723; doi:10.1186/s12864-021-08284-9)
Supplement: Supplementary file 1 — Additional file 1. [file 12864_2021_8284_MOESM1_ESM.zip › Figure S. file/Fig.S2.docx]

**
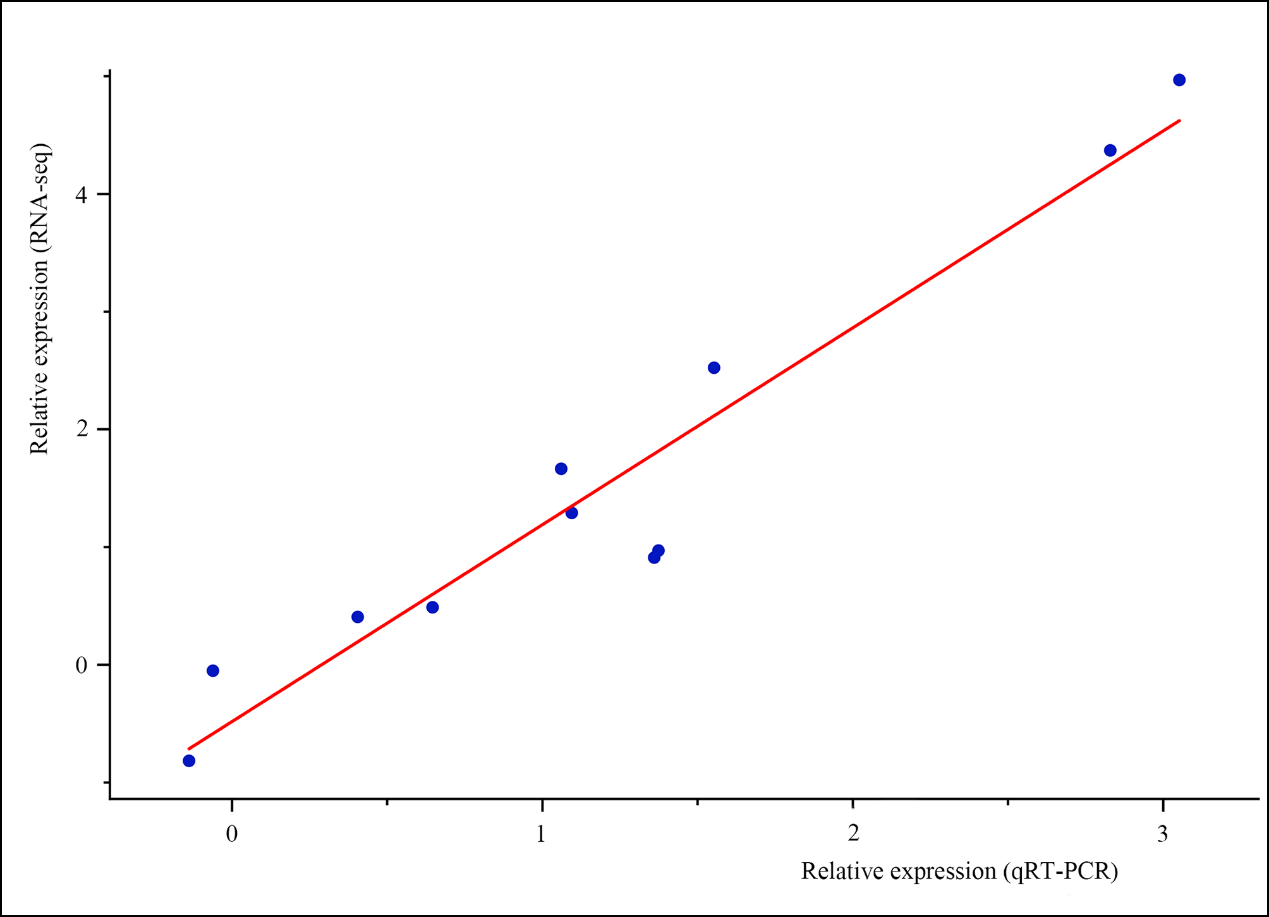
**

**Fig. S2 |** Verification of RNA-seq results by qRT-PCR. The *x*-axis and *y*-axis represent the relative gene expression levels analyzed independently by qRT-PCR and RNA-seq, respectively. The 11 key DEGs are *CL8627.Contig6_All*, *CL1983.Contig1_All*, *CL456.Contig3_All*, *CL652.Contig1_All*, *CL6704.Contig1_All*, *CL4410.Contig1_All*, *CL2996.Contig9_All*, *CL118.Contig41_All*, *CL3812.Contig6_All*, *CL3209.Contig8_All*, and *CL3274.Contig2_All*, respectively
