# Supplementary figures and images for: Transcriptome analysis of Auricularia fibrillifera fruit-body responses to drought stress and rehydration
Source: BMC Genomics. 2022 Jan 15;23:58. doi: 10.1186/s12864-021-08284-9 (PMC8760723; doi:10.1186/s12864-021-08284-9)

**
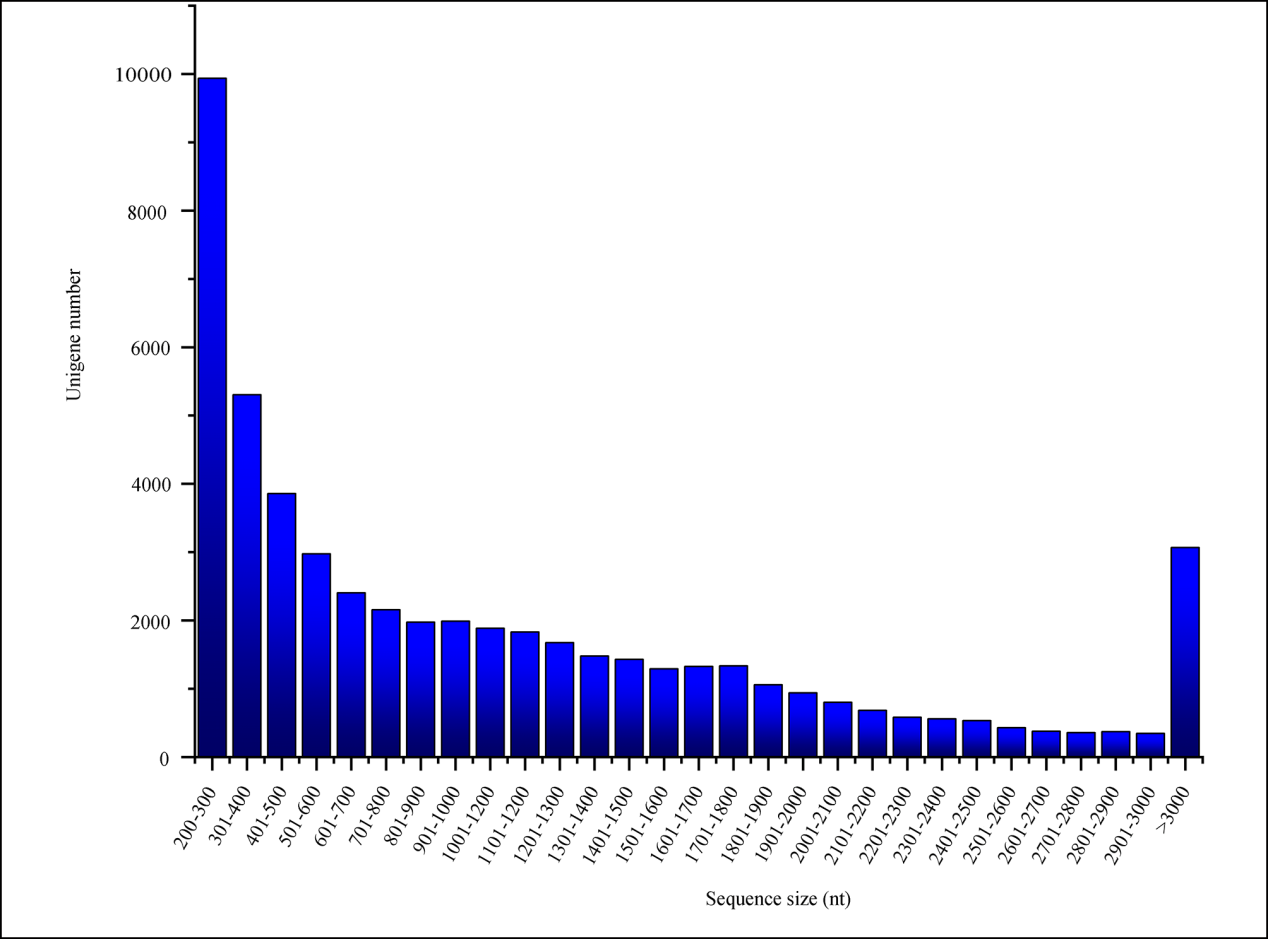
**

**Fig. S1 |** Distribution of unigene lengths

Supplement: Supplementary file 1 — Additional file 1. [file 12864_2021_8284_MOESM1_ESM.zip › Figure S. file/Fig.S1.docx]

**
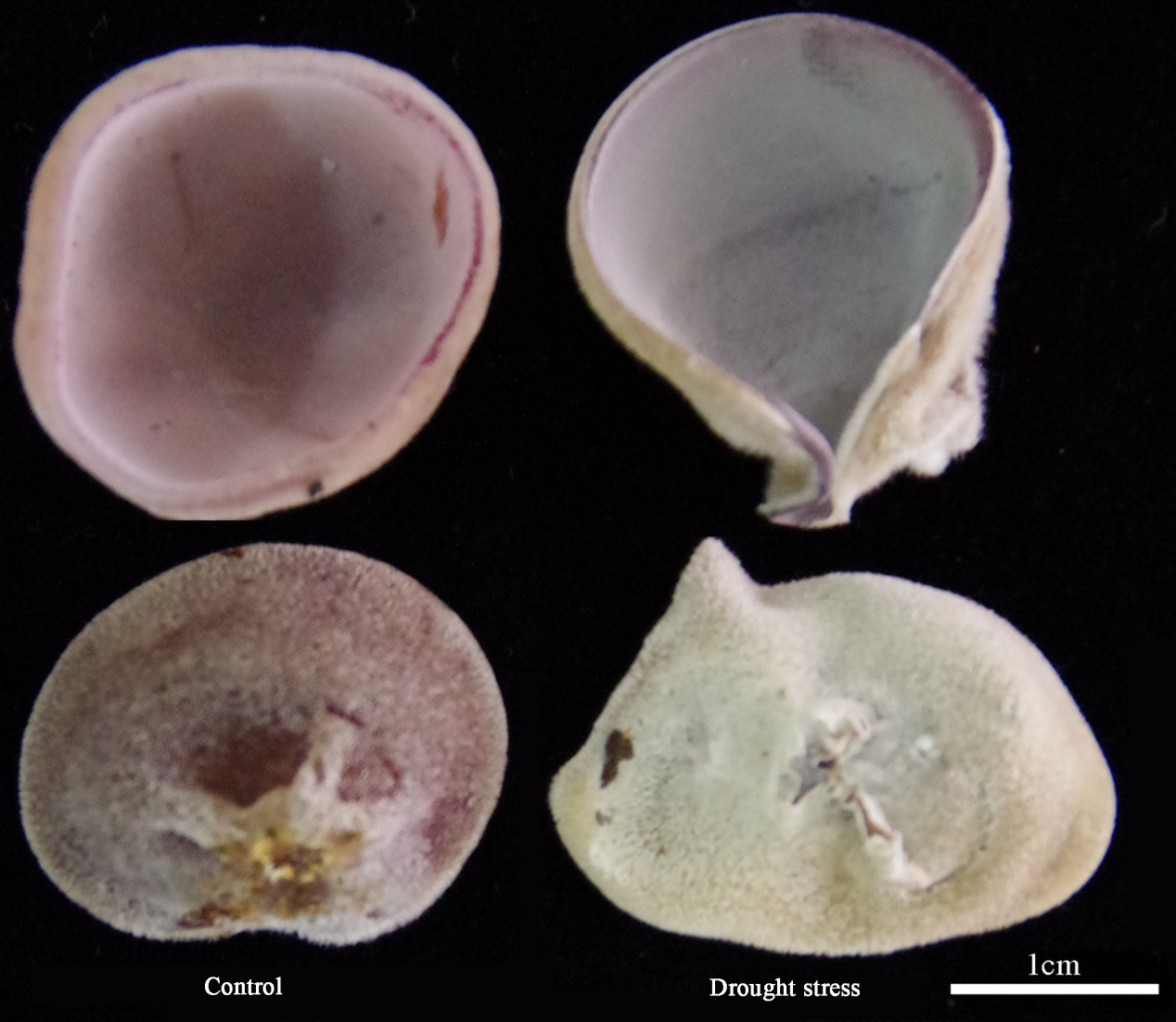
**

**Fig. S3 |** The shrinking fruiting bodies under drought-stress conditions

Supplement: Supplementary file 1 — Additional file 1. [file 12864_2021_8284_MOESM1_ESM.zip › Figure S. file/Fig.S3.docx]
