# Supplementary material for: Transcriptome analysis of Auricularia fibrillifera fruit-body responses to drought stress and rehydration
Source: BMC Genomics. 2022 Jan 15;23:58. doi: 10.1186/s12864-021-08284-9 (PMC8760723; doi:10.1186/s12864-021-08284-9)
Supplement: Supplementary file 2 — Additional file 2. [file 12864_2021_8284_MOESM2_ESM.zip › Table S/Table S1.docx]

**Table S1**  Coding sequence statistics of *A. fibrillifera*

| Total number of coding sequences | | 37,530 |
| --- | --- | --- |
|  | Total length (bp) | 41,066,277 |
|  | Scaffold N50 size (bp) | 1,425 |
|  | Scaffold N90 size (bp) | 531 |
|  | Max length (bp) | 11,805 |
|  | Min length (bp) | 297 |
|  | GC (%) | 62.04 |
