# Supplementary material for: Transcriptome analysis of Auricularia fibrillifera fruit-body responses to drought stress and rehydration
Source: BMC Genomics. 2022 Jan 15;23:58. doi: 10.1186/s12864-021-08284-9 (PMC8760723; doi:10.1186/s12864-021-08284-9)
Supplement: Supplementary file 2 — Additional file 2. [file 12864_2021_8284_MOESM2_ESM.zip › Table S/Table S2.docx]

**Table S2** Significantly enriched GO terms

| **Stages** | | **DEG number** | **GO Term ID** | **GO Terms** | **Percentage** | **Q value** |
| --- | --- | --- | --- | --- | --- | --- |
| Drought stress | Up | 4996 | GO:0004707 | MAP kinase activity | 0.26% | 2.70E^−06^ |
|  |  |  | GO:0005739 | Mitochondrion | 0.82% | 1.34E^−03^ |
|  |  |  | GO:0003847 | 1-alkyl-2-acetylglycerophosphocholine esterase activity | 0.10% | 1.82E^−03^ |
|  |  |  | GO:0004129 | Cytochrome-c oxidase activity | 0.40% | 1.82E^−03^ |
|  |  |  | GO:0008289 | Lipid binding | 0.20% | 1.82E^−03^ |
|  |  |  | GO:0031505 | Fungal-type cell wall organization | 0.18% | 4.05E^−03^ |
|  |  |  |  |  |  |  |
|  | Down | 9239 | GO:0003735 | Structural constituent of ribosome | 5.50% | 4.78E^−14^ |
|  |  |  | GO:0006412 | Translation | 5.09% | 2.98E^−12^ |
|  |  |  | GO:0005840 | Ribosome | 4.07% | 3.73E^−07^ |
|  |  |  | GO:0003924 | GTPase activity | 1.28% | 1.80E^−04^ |
|  |  |  | GO:0005525 | GTP binding | 1.43% | 8.41E^−04^ |
|  |  |  |  |  |  |  |
| Rehydration | Up | 4373 | GO:0006950 | Response to stress | 0.59% | 1.65E^−05^ |
|  |  |  | GO:0004707 | MAP kinase activity | 0.23% | 8.92E^−04^ |
|  |  |  | GO:0022857 | Transmembrane transporter activity | 0.23% | 2.87E^−03^ |
|  |  |  |  |  |  |  |
|  | Down | 4166 | GO:0003735 | Structural constituent of ribosome | 6.82% | 1.27E^−20^ |
|  |  |  | GO:0006412 | Translation | 6.34% | 1.27E^−20^ |
|  |  |  | GO:0005840 | Ribosome | 5.23% | 1.94E^−16^ |
|  |  |  | GO:0003746 | Translation elongation factor activity | 0.86% | 2.74E^−04^ |
|  |  |  | GO:0030245 | Cellulose catabolic process | 0.14% | 1.83E^−03^ |
