# Supplementary material for: Transcriptome analysis of Auricularia fibrillifera fruit-body responses to drought stress and rehydration
Source: BMC Genomics. 2022 Jan 15;23:58. doi: 10.1186/s12864-021-08284-9 (PMC8760723; doi:10.1186/s12864-021-08284-9)
Supplement: Supplementary file 2 — Additional file 2. [file 12864_2021_8284_MOESM2_ESM.zip › Table S/Table S3.docx]

**Table S3** Key pathways and DEGs under drought-stress conditions

| **Pathways** | **Gene ID** | **Log_2_FC** | **Q value** |
| --- | --- | --- | --- |
| Phagosome | *CL118.Contig41_All* | 9.35 | 2.32E^−59^ |
|  | *Unigene13790_All* | 5.02 | 2.31E^−05^ |
|  | *CL415.Contig8_All* | 4.34 | 7.97E^−04^ |
|  | *Unigene1180_All* | 4.08 | 8.42E^−41^ |
|  | *Unigene14821_All* | 3.86 | 2.00E^−16^ |
|  | *CL652.Contig1_All* | 3.64 | 6.05E^−09^ |
|  | *Unigene13168_All* | 3.29 | 1.96E^−17^ |
|  | *CL4156.Contig1_All* | −2.67 | 8.49E^−17^ |
|  | *CL4156.Contig2_All* | −4.03 | 5.94E^−12^ |
|  | *CL4156.Contig3_All* | −4.50 | 3.74E^−04^ |
|  |  |  |  |
|  | *Unigene14313_All* | 6.19 | 3.95E^−10^ |
|  | *Unigene14272_All* | 5.66 | 1.44E^−07^ |
|  | *Unigene13787_All* | 4.92 | 4.13E^−05^ |
|  | *CL7824.Contig2_All* | 4.72 | 1.33E^−04^ |
| Peroxisome | *Unigene11360_All* | 4.66 | 1.40E^−35^ |
|  | *Unigene13493_All* | 4.48 | 5.17E^−31^ |
|  | *Unigene14552_All* | 4.48 | 1.47E^−06^ |
|  | *Unigene13779_All* | 4.02 | 5.17E^−05^ |
|  | *CL6767.Contig1_All* | 3.84 | 3.54E^−247^ |
|  | *Unigene14581_All* | 3.26 | 6.20E^−12^ |
|  |  |  |  |
| Caffeine metabolism | *CL2727.Contig6_All* | 1.27 | 1.12E^−08^ |
|  | *CL2727.Contig3_All* | −1.25 | 7.44E^−05^ |
|  | *CL2727.Contig1_All* | −1.64 | 7.45E^−118^ |
|  | *CL2727.Contig7_All* | −1.70 | 6.29E^−20^ |
|  | *CL2727.Contig2_All* | −1.87 | 2.84E^−160^ |
|  |  |  |  |
|  | *CL504.Contig14_All* | 4.71 | 1.40E^−04^ |
|  | *Unigene11156_All* | 3.88 | 1.78E^−70^ |
|  | *CL414.Contig4_All* | 3.50 | 3.11E^−15^ |
|  | *Unigene219_All* | 3.41 | 0 |
|  | *CL414.Contig2_All* | 3.38 | 2.50E^−80^ |
|  | *Unigene13579_All* | 3.37 | 4.24E^−11^ |
| Tyrosine metabolism | *Unigene12623_All* | 3.30 | 9.20E^−09^ |
|  | *Unigene14256_All* | 2.82 | 5.98E^−16^ |
|  | *CL504.Contig12_All* | 2.66 | 6.36E^−71^ |
|  | *Unigene10928_All* | −6.86 | 6.06E^−15^ |
|  |  |  |  |
| Proline and arginine metabolism | *CL1716.Contig2_All* | 7.72 | 2.35E^−25^ |
|  | *Unigene13724_All* | 6.92 | 9.55E^−08^ |
|  | *CL7737.Contig3_All* | 6.21 | 3.45E^−147^ |
|  | *Unigene12633_All* | 5.82 | 6.46E^−06^ |
|  | *CL868.Contig6_All* | 5.54 | 7.38E^−02^ |
|  | *Unigene14459_All* | 4.54 | 1.18E^−80^ |
|  | *Unigene4554_All* | 4.19 | 1.74E^−11^ |
|  | *CL2495.Contig4_All* | 3.20 | 1.39E^−12^ |
|  | *CL183.Contig3_All* | 2.71 | 1.54E^−19^ |
|  | *Unigene14388_All* | 2.06 | 1.42E^−08^ |
|  |  |  |  |
| Alanine, aspartate and glutamate metabolism | *Unigene5564_All* | 6.30 | 9.64E^−02^ |
|  | *Unigene14099_All* | 6.08 | 5.54E^−17^ |
|  | *Unigene15963_All* | 6.04 | 1.73E^−16^ |
|  | *CL3057.Contig3_All* | 5.60 | 0 |
|  | *CL3057.Contig1_All* | 5.44 | 6.61E^−304^ |
|  | *CL3057.Contig4_All* | 4.74 | 0 |
|  | *Unigene3119_All* | 4.15 | 2.95E^−90^ |
|  | *Unigene3813_All* | 4.12 | 0 |
|  | *Unigene16234_All* | 4.02 | 3.64E^−08^ |
|  | *Unigene6119_All* | 3.54 | 0 |
|  |  |  |  |
| Cysteine and methionine metabolism | *CL8066.Contig1_All* | 6.02 | 3.26E^−09^ |
|  | *Unigene12602_All* | 5.60 | 2.49E^−07^ |
|  | *Unigene7556_All* | 5.41 | 1.33E^−06^ |
|  | *Unigene14235_All* | 5.10 | 1.39E^−09^ |
|  | *CL2016.Contig1_All* | 5.05 | 7.92E^−252^ |
|  | *Unigene11810_All* | 4.72 | 1.33E^−04^ |
|  | *Unigene8964_All* | 4.48 | 4.37E^−04^ |
|  | *Unigene12615_All* | 4.19 | 2.04E^−09^ |
|  | *Unigene14234_All* | 3.85 | 6.58E^−31^ |
|  | *Unigene13579_All* | 3.37 | 4.24E^−11^ |
|  |  |  |  |
| Glycine, serine and threonine metabolism | *CL2016.Contig1_All* | 5.05 | 7.92E^−252^ |
|  | *Unigene14459_All* | 4.54 | 1.53E^−94^ |
|  | *Unigene14157_All* | 4.34 | 7.97E^−04^ |
|  | *CL9001.Contig1_All* | 4.04 | 0 |
|  | *CL7450.Contig2_All* | 3.31 | 0 |
|  | *Unigene9472_All* | 3.07 | 9.20E^−196^ |
|  | *Unigene1009_All* | 2.68 | 1.81E^−04^ |
|  | *CL7710.Contig3_All* | 2.65 | 0 |
|  | *Unigene11002_All* | 2.05 | 8.06E^−12^ |
|  | *CL9001.Contig2_All* | 2.00 | 0 |
|  |  |  |  |
| Histidine metabolism | *Unigene14459_All* | 4.54 | 1.53E^−94^ |
|  | *CL414.Contig4_All* | 3.50 | 3.11E^−15^ |
|  | *CL414.Contig2_All* | 3.38 | 2.50E^−80^ |
|  | *CL2495.Contig4_All* | 3.20 | 4.36E^−37^ |
|  | *CL183.Contig3_All* | 2.71 | 5.59E^−18^ |
|  | *CL106.Contig3_All* | 2.50 | 3.20E^−09^ |
|  | *CL5511.Contig4_All* | 2.08 | 1.31E^−34^ |
|  | *CL5511.Contig2_All* | 1.88 | 7.48E^−05^ |
|  | *CL183.Contig20_All* | 1.57 | 9.86E^−18^ |
|  | *CL414.Contig8_All* | 1.40 | 4.43E^−05^ |
|  |  |  |  |
| Lysine degradation | *Unigene14459_All* | 4.54 | 1.53E^−94^ |
|  | *CL2495.Contig4_All* | 3.20 | 4.36E^−37^ |
|  | *CL183.Contig3_All* | 2.71 | 5.59E^−18^ |
|  | *CL7710.Contig3_All* | 2.65 | 0 |
|  | *CL7987.Contig2_All* | 2.30 | 0 |
|  | *CL8386.Contig3_All* | 2.00 | 2.16E^−246^ |
|  | *CL8386.Contig2_All* | 1.67 | 1.51E^−81^ |
|  | *CL183.Contig20_All* | 1.57 | 9.86E^−18^ |
|  | *CL1893.Contig7_All* | 1.38 | 1.28E^−15^ |
|  | *Unigene3676_All* | 1.30 | 2.41E^−09^ |
|  |  |  |  |
| Tryptophan metabolism | *CL1716.Contig2_All* | 7.72 | 5.00E^−24^ |
|  | *Unigene13724_All* | 6.92 | 2.41E^−15^ |
|  | *CL7737.Contig3_All* | 6.21 | 1.69E^−173^ |
|  | *Unigene12633_All* | 5.82 | 6.87E^−15^ |
|  | *CL868.Contig6_All* | 5.54 | 4.20E^−07^ |
|  | *CL7824.Contig2_All* | 4.72 | 1.33E^−04^ |
|  | *Unigene14459_All* | 4.54 | 1.53E^−94^ |
|  | *CL5445.Contig1_All* | 4.42 | 5.56E^−04^ |
|  | *Unigene4554_All* | 4.19 | 5.03E^−21^ |
|  | *Unigene14581_All* | 3.26 | 6.20E^−12^ |
|  |  |  |  |
| Valine, leucine and isoleucine degradation | *CL1142.Contig1_All* | 6.25 | 7.17E^−161^ |
|  | *Unigene14459_All* | 4.54 | 1.53E^−94^ |
|  | *CL6767.Contig1_All* | 3.84 | 3.54E^−247^ |
|  | *CL5904.Contig1_All* | 3.30 | 5.46E^−38^ |
|  | *CL2495.Contig4_All* | 3.20 | 4.36E^−37^ |
|  | *Unigene3361_All* | 3.14 | 1.84E^−24^ |
|  | *CL5904.Contig2_All* | 2.78 | 7.66E^−127^ |
|  | *CL183.Contig3_All* | 2.71 | 5.59E^−18^ |
|  | *CL4794.Contig2_All* | 1.87 | 4.02E^−21^ |
|  | *CL2495.Contig1_All* | −7.50 | 1.61E^−21^ |
|  |  |  |  |
| Fructose and mannose metabolism | *CL3093.Contig2_All* | 3.89 | 0 |
|  | *Unigene12980_All* | 3.89 | 9.89E^−11^ |
|  | *Unigene15955_All* | 3.82 | 1.70E^−04^ |
|  | *Unigene14606_All* | 3.81 | 4.83E^−27^ |
|  | *Unigene10303_All* | 2.98 | 9.59E^−60^ |
|  | *CL8001.Contig1_All* | 2.80 | 1.14E^−170^ |
|  | *CL2479.Contig4_All* | 2.74 | 3.52E^−90^ |
|  | *CL3164.Contig2_All* | 2.73 | 0 |
|  |  |  |  |
| Mannose type O-glycan biosynthesis | *CL8620.Contig2_All* | 7.43 | 2.17E^−20^ |
|  | *CL2554.Contig2_All* | 6.46 | 7.53E^−02^ |
|  | *CL2554.Contig4_All* | 5.80 | 3.38E^−08^ |
|  | *CL2554.Contig1_All* | 5.54 | 4.43E^−07^ |
|  | *Unigene12925_All* | 5.34 | 4.64E^−11^ |
|  | *CL2554.Contig3_All* | 5.24 | 4.87E^−06^ |
|  | *Unigene16348_All* | 5.19 | 4.45E^−10^ |
|  | *Unigene11486_All* | 5.10 | 1.39E^−09^ |
|  | *Unigene16378_All* | 4.57 | 3.19E^−12^ |
|  | *Unigene20984_All* | −6.16 | 4.27E^−10^ |
|  |  |  |  |
| Amino sugar and nucleotide sugar metabolism | *Unigene104_All* | 10.15 | 0 |
|  | *Unigene5550_All* | 9.71 | 4.80E^−196^ |
|  | *CL8307.Contig2_All* | 9.62 | 1.11E^−68^ |
|  | *CL3209.Contig8_All* | 9.29 | 0 |
|  | *CL1874.Contig4_All* | 8.68 | 1.55E^−48^ |
|  | *Unigene13034_All* | 8.65 | 2.96E^−40^ |
|  | *Unigene14521_All* | 7.91 | 1.51E^−26^ |
|  | *CL1706.Contig1_All* | 7.60 | 2.84E^−02^ |
|  | *CL1129.Contig6_All* | 7.11 | 3.58E^−14^ |
|  | *CL3098.Contig3_All* | 6.70 | 4.31E^−25^ |
|  |  |  |  |
| MAPK signaling pathway - yeast | *Unigene9338_All* | 10.52 | 2.38E^−111^ |
|  | *Unigene104_All* | 10.15 | 0 |
|  | *CL1874.Contig4_All* | 8.68 | 3.39E^−41^ |
|  | *Unigene13034_All* | 8.65 | 1.52E^−40^ |
|  | *CL8655.Contig6_All* | 7.65 | 3.98E^−23^ |
|  | *Unigene5387_All* | 6.90 | 5.36E^−15^ |
|  | *CL6689.Contig2_All* | 6.85 | 1.07E^−14^ |
|  | *CL3017.Contig2_All* | 6.44 | 1.07E^−11^ |
|  | *Unigene2205_All* | 6.44 | 1.76E^−11^ |
|  | *CL3858.Contig3_All* | 4.75 | 1.12E^−04^ |
