# Supplementary material for: Transcriptome analysis of Auricularia fibrillifera fruit-body responses to drought stress and rehydration
Source: BMC Genomics. 2022 Jan 15;23:58. doi: 10.1186/s12864-021-08284-9 (PMC8760723; doi:10.1186/s12864-021-08284-9)
Supplement: Supplementary file 2 — Additional file 2. [file 12864_2021_8284_MOESM2_ESM.zip › Table S/Table S4.docx]

**Table S4** Key pathways and DEGs during rehydration process

| **Pathways** | **Gene ID** | **log_2_FC** | **Q value** |
| --- | --- | --- | --- |
| Diterpenoid biosynthesis | *Unigene8009_All* | 3.73 | 8.00E^−13^ |
|  | *CL4791.Contig4_All* | 3.34 | 5.76E^−28^ |
|  | *CL8530.Contig2_All* | 2.50 | 2.18E^−27^ |
|  | *CL8989.Contig6_All* | 2.13 | 6.80E^−18^ |
|  | *CL4791.Contig1_All* | 1.73 | 3.52E^−161^ |
|  | *CL3355.Contig5_All* | 1.72 | 4.58E^−09^ |
|  | *CL264.Contig8_All* | 1.51 | 4.84E^−06^ |
|  | *CL4791.Contig5_All* | 1.50 | 4.14E^−87^ |
|  | *CL4791.Contig3_All* | 1.45 | 4.83E^−11^ |
|  | *CL5200.Contig3_All* | −1.72 | 2.93E^−36^ |
|  |  |  |  |
| Butanoate metabolism | *Unigene5564_All* | 5.79 | 5.68E^−08^ |
|  | *CL3057.Contig1_All* | 4.89 | 1.66E^−137^ |
|  | *CL1028.Contig1_All* | 4.58 | 9.20E^−93^ |
|  | *CL3057.Contig4_All* | 3.79 | 0 |
|  | *Unigene3125_All* | 3.31 | 2.67E^−02^ |
|  | *CL3057.Contig3_All* | 3.24 | 0 |
|  | *Unigene6119_All* | 2.52 | 0 |
|  | *CL1028.Contig2_All* | 2.49 | 0 |
|  | *Unigene3813_All* | 2.20 | 5.29E^−284^ |
|  | *CL7987.Contig2_All* | 1.43 | 0 |
|  |  |  |  |
| C_5_-branched dibasic acid metabolism | *CL4410.Contig1_All* | 1.27 | 0 |
|  | *CL4410.Contig2_All* | 1.24 | 0 |
|  | *CL6195.Contig1_All* | −1.39 | 4.87E^−20^ |
|  | *CL7598.Contig2_All* | 1.84 | 9.24E^−279^ |
|  | *CL7598.Contig3_All* | 1.09 | 1.61E^−98^ |
|  | *CL8753.Contig2_All* | 1.05 | 3.65E^−55^ |
