# Supplementary material for: Transcriptome analysis of Auricularia fibrillifera fruit-body responses to drought stress and rehydration
Source: BMC Genomics. 2022 Jan 15;23:58. doi: 10.1186/s12864-021-08284-9 (PMC8760723; doi:10.1186/s12864-021-08284-9)
Supplement: Supplementary file 2 — Additional file 2. [file 12864_2021_8284_MOESM2_ESM.zip › Table S/Table S5.docx]

**TABLE S5 |** qPCR primers for DEGs in *A. fibrillifera* and the internal control gene

| **Gene ID** | **Forward primers** | **Reverse primers** | **Annealing temperature (**°C**)** |
| --- | --- | --- | --- |
| *CL4410.Contig1_All* | TGGCTCGGGTAGTTGTAGTG | GCGATTGTATTGAGAAGCCCA | 59.0 |
| *CL3812.Contig6_All* | CTTCGCCCATTTGTCGCTAA | CAATCAGGACCTTGAGCAGC | 59.0 |
| *CL2996.Contig9_All* | GCTCTCAACCGCCCTACTTA | CGCAGCTCATGGTAAGATCG | 59.0 |
| *CL3209.Contig8_All* | GCGTCCATCTCTACCTCCTC | CTCGTTGCCATTGACACCAG | 59.5 |
| *CL3274.Contig2_All* | ACCACTGGTCGTCGTTAAGT | CGCAAGTCAAAGCCCAAGAA | 59.5 |
| *CL1983.Contig1_All* | CTCTTCATCCTCGACGACCA | GCCGATCCGTTCATCCAATC | 59.0 |
| *CL8627.Contig6_All* | CGACAAGCTGAAACCGACAA | CACATCGAACGTGGAAGTCC | 59.0 |
| *Unigene5564_All* | AGGCCCAGAAAGAAGTCGAA | TTTCCGTGAGCCTTTTGAGC | 59.0 |
| *CL118.Contig41_All* | TGGACTCTGGTGATGGTGTC | AGTAGTCGGTCAAATCGCGA | 59.0 |
| *CL6704.Contig1_All* | GCATACGCCACTGACAAGAC | GCTGCCGTTGTTTGTTGTTC | 59.0 |
| *CL652.Contig1_All* | CTTGGAGGTTGTTGCAGGTC | CAATTGACGATGGCTTTGCG | 58.5 |
| *CL456.Contig3_All* | AGCGGTTGGATTGAGATTGC | TACTCCTTGTTGCCGTCCTT | 59.0 |
| *GAPDH* | GCATCGGGCGGATTGTGA | GCTTGCCGTCCTTGGTCT | 53.0 |
